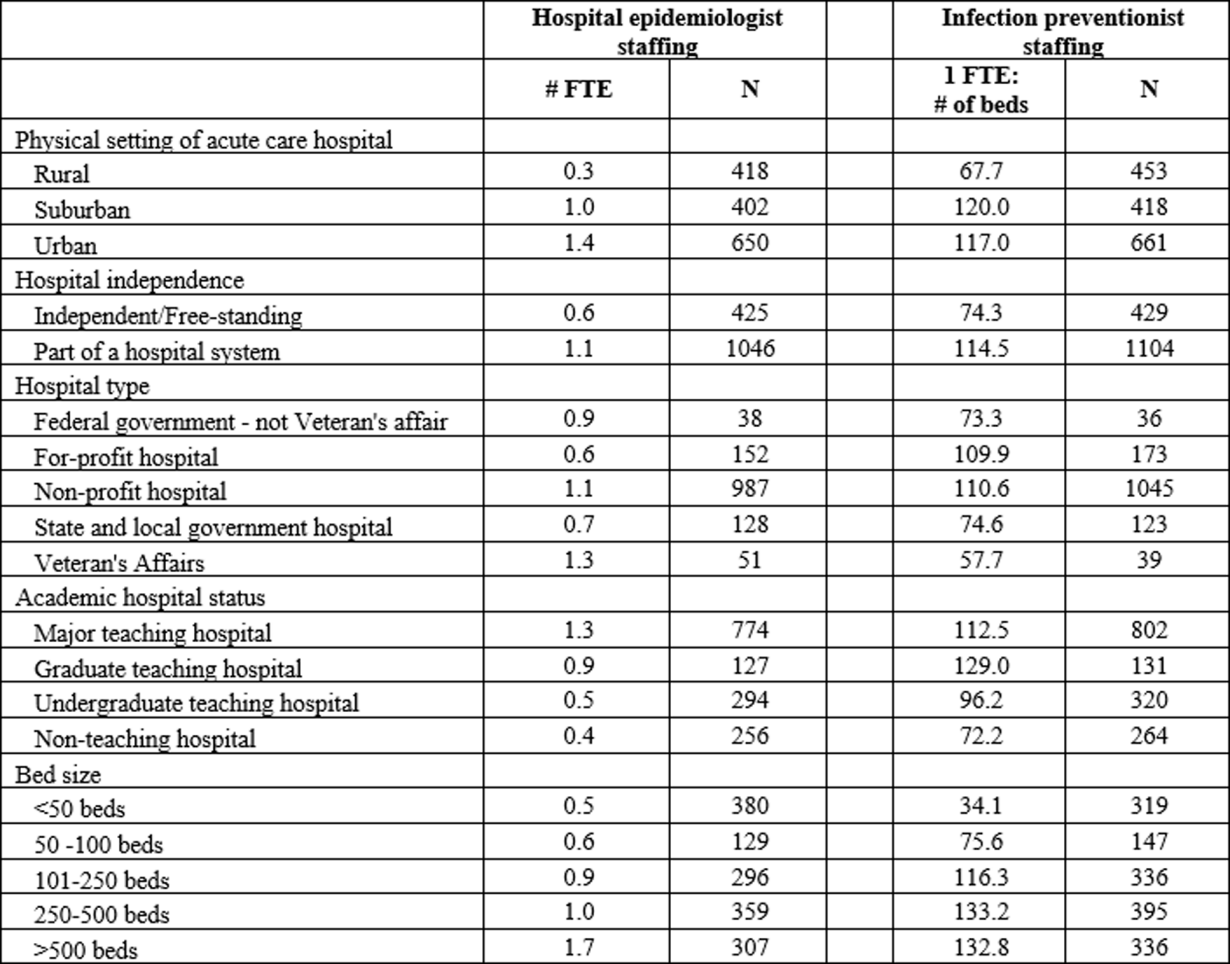# 277 Performance Improvement in Healthcare Facility Measles Management

**DOI:** 10.1017/ash.2026.10639

**Published:** 2026-06-23

**Authors:** Sara Reese, Becca Crapanzano-Sigafoos

**Affiliations:** 1 APIC

## Abstract

**Background:** Recently, the Society for Healthcare Epidemiologists of America (SHEA) and the Association for Professionals in Infection Control and Epidemiology (APIC) have released full time equivalent (FTE) staffing recommendations for hospital epidemiologists (HE) (at least 0.3 FTE for 0-50 beds, 0.5 FTE for 51-300 beds, 1.0 FTE for <300 beds) and infection preventionists (IP)(between 1 IP: 69 beds and 1 IP: 100 beds depending on size and patient acuity) for acute care hospitals. As these important roles become more complex for a variety of reasons, the necessity for appropriate staffing is becoming more apparent. The purpose of this project was to utilize data from the 2025 APIC MegaSurvey to describe current staffing for HEs and IPs. **Methods:** The 2025 APIC MegaSurvey was distributed to over 13,000 members and shared through email, QR codes at the APIC national conference and social media campaigns from June 6 through July 31, 2025. The respondents provided the number of FTEs for HE and IPs in acute care hospitals along with hospital characteristics including bed size, physical setting, academic affiliation, ownership status, and hospital type. The average FTEs for HE (more FTEs = more HE support) and the 1 FTE: per number of beds ratio (lower the ratio = more IP support) for IPs were calculated and stratified by the hospital characteristics. **Results:** There were 1,471 respondents who reported the FTEs for HE with an average of 0.97 FTE. The FTE IP: number of beds reported for the 1,533 respondents was 1 FTE:103.3 beds. Rural hospitals reported the lowest FTE for HE (0.3, n=418) and yet had the lowest 1 FTE: number of beds ratio (1:67.7, n=453). Smaller hospitals (<50 beds) reported the lowest FTE for HE (0.5, n=380) with the lowest 1 FTE: number of beds ratio (1:34.1, n=319). As the hospitals increased in size, the FTE for HE increased with 1.7 FTE for hospitals <500 beds (n=307) but also reported the highest 1 FTE: number of beds ratio of 1:132.8 (n=336). **Conclusions:** The data suggest that staffing for HE and IPs are impacted by numerous factors including bed size, ownership, academic affiliation and physical location. The dyad model that includes the HE and IP leaders improves communication, collaboration, and successful attainment of institutional goals. Future efforts should focus on determining the positive impacts of appropriate staffing utilizing the dyad model.

**Figure:**